# The Strengths of People in Low-SES Positions: An Identity-Reframing Intervention Improves Low-SES Students’ Achievement Over One Semester

**DOI:** 10.1177/19485506241284806

**Published:** 2024-10-20

**Authors:** Christina A. Bauer, Gregory Walton, Veronika Job, Nicole Stephens

**Affiliations:** 1 University of Vienna, Austria; 2 Stanford University, CA, USA; 3 Northwestern University, Evanston, IL, USA

**Keywords:** socioeconomic status, first-generation college students, identity-reframing, educational inequality, empowerment

## Abstract

Students from low-socioeconomic status (SES) backgrounds such as first-generation or low-income students are often portrayed as deficient, lacking in skills and potential to succeed at university. We hypothesized that such representations lead low-SES students to see their SES-identity as a barrier to success and impair achievement. If so, reframing low-SES students’ identity as a source of strength may help them succeed. Testing this hypothesis in a highly scalable form, we developed an online low-SES-identity-reframing exercise. In Experiment 1 (*N* = 214), this exercise helped low-SES students to see their SES-identity more as a source of success and boosted their performance on an academic task by 13%. In Experiment 2, a large randomized-controlled intervention field experiment (*N* = 786), we implemented the identity-reframing intervention in a university’s online learning program. This improved low-SES students’ grades over the semester. Recognizing the strengths low-SES students bring to university can help students access these strengths and apply them to schooling.

Students from lower socioeconomic status (SES) backgrounds such as first-generation or low-income students face many challenges to succeeding at university (Bourdieu, 1984; Goudeau et al., 2024; Mehta et al., 2011). For instance, first-generation students may have less access to the types of cultural capital that helps students succeed in universities (e.g., knowledge about university or classic literature). Low-income students may have less economic capital (e.g., money for expenses). Since many students are both first-generation and low-income students, many face both kinds of low-SES–based challenges.

In contending with low-SES–based challenges (e.g., working extra jobs; learning about university-culture), low-SES students often show and develop strengths (Frankenhuis & Nettle, 2020; Kraus et al., 2010). These strengths, however, may go unrecognized. Instead, low-SES students are commonly represented as deficient, lacking in the skills and potential needed to succeed at university (Durante & Fiske, 2017). *The Atlantic*, for example, proclaims “First-Generation College-Goers: Unprepared and Behind” (Riggs, 2014). *Science* magazine reports, “Low-income-students lose ground” (Avery et al., 2020). There are certainly inequalities in achievement outcomes between low- and high-SES students. Yet, when students are labeled as “behind,” or “losing ground,” without explicating the disadvantages that cause these inequalities, deficiency is presented as a defining characteristic of their group (Belmi et al., 2023; McKay & Devlin, 2016).

We theorize that this stigmatizing representation can produce self-fulfilling effects. It may obscure students’ strengths even to themselves, undermining low-SES students’ academic confidence (see Bauer et al., 2023), and impairing their performance. If so, inverting this narrative may help low-SES students succeed. To examine this possibility, we created an intervention that reframes low-SES students’ identity as a source of strength and agency rather than weakness and deficiency. We tested the effects of this low-SES-identity-reframing intervention on students’ self-views and achievement. Through reading-and-writing exercises, the intervention specifically highlights (a) how facing challenges that arise from lower-SES backgrounds can lead people to develop strengths such as perseverance or resilience and (b) how students can apply these strengths to succeed at their university. After reading stories about other low-SES students’ experiences, participating students reflect on the strengths *they* have shown and developed in response to low-SES–based challenges, and how they can use these strengths to succeed at their university. In changing the representation of an identity, this approach complements research which has sought to change the representation of success in ways that are more compatible with diverse identities (e.g., Kray et al., 2002).

To develop the low-SES-identity-reframing intervention, we drew on an identity-reframing intervention for students with refugee backgrounds (Bauer et al., 2021). That exercise highlighted challenges refugees experienced, such as escaping their home country and seeking refuge elsewhere, and how these challenges can confer strengths that refugees can use to succeed academically. Implemented in an online-university dedicated to refugees, this refugee-identity-reframing intervention boosted refugees’ academic engagement over a year.

Could this approach be helpful more broadly, even for students in a very different context? Refugees have faced acute challenges, often including life-endangering threats. Academic challenges may seem trivial by comparison. A strong-and-capable identity may hence be relatively accessible for refugees. By contrast, low-SES students typically face chronic challenges, which only become more pressing as students enter university. In developing the identity-reframing intervention for low-SES students, we test not only whether low-SES students can benefit from this approach but also whether identity-reframing could have benefits more generally for stigmatized student groups beyond refugees. In addition, extending the focus of the prior research on academic engagement, here we test effects on achievement, including fine-grained weekly performance data over a semester. Given that the goal of schooling is to promote learning and achievement, this outcome is important.

In applying the identity-reframing approach to low-SES students, we also extend prior research aiming to support low-SES students’ academic success.

First, in testing causal effects on long-term real-world performance, we go beyond research, which found encouraging short-term effects of highlighting low-SES students’ strengths. This past research invited low-SES students to broadly reflect on strengths they had based on their SES background, including strengths such as family and community resources, not just strengths students show in contending with low-SES–based challenges (as is the focus of identity-reframing interventions). As compared to a randomized control, this led students to see themselves as more of an asset to their school and to report they would be more likely to persevere in the face of academic difficulty (Hernandez et al., 2021). However, while these self-reports correlated with end-of-term grades, this research did not find any causal effects on grades or other behavioral outcomes. In section “General Discussion,” we will discuss differences in the identity-reframing approach that may contribute to its long-term effects.

The low-SES-reframing intervention also extends previous research on the *difference—education intervention—*an intervention that has been shown to raise achievement among first-generation students (Stephens et al., 2014). The difference-education intervention conveys that students from low- and high-SES backgrounds can both succeed in university, but simply need different strategies to do so, given their unique backgrounds. The identity-reframing intervention takes a simpler and more direct approach to mitigate stigma. It highlights the strengths low-SES students have shown in contending with low-SES–based challenges.

## The Present Research

We report two randomized-controlled experiments. Experiment 1 examined immediate effects on low-SES students’ representation of their SES-identity and their performance on an academic task. In Experiment 2, a pre-registered longitudinal randomized-controlled field experiment, we integrated the intervention into an online biology program, a component of a traditional in-person university course, and examined effects on students’ grades in the program over a semester. Our main hypotheses were that identity-reframing would improve low-SES students’ view of their SES-identity (Experiment 1), academic confidence (Experiment 2), and immediate- and long-term performance (Experiments 1 and 2, respectively).

We designed the low-SES-reframing intervention for both first-generation- and low-income students, taking into account both students’ socio-cultural and economic backgrounds as different facets of SES (American Psychological Association, n.d.). Accordingly, we test effects among first-generation (Experiments 1 and 2) and low-income students (Experiment 2 only). Exploratory analyses in Experiment 2 test for any differences in effects across low-SES subgroups.

All materials, data, and code are available at https://osf.io/gw6s9/?view_only=9a925b004ce6462882bc83c723181a84. Experiment 2 is pre-registered at https://aspredicted.org/sx6xb.pdf.

## Experiment 1: Immediate Effects on Students’ Representation of Their SES-Identity and Academic Performance

In Experiment 1, we developed the low-SES-identity-reframing intervention and tested its immediate effects with 214 first-generation students at a large U.S. university. Main outcomes were the extent to which students saw strengths and challenges in their SES-identity and their performance on an academic task.

### Method

#### Participants

We recruited first-generation students through a university-registrar’s email list that included all first- and second-year first-generation students at the university. To ensure that only these first-generation students participated, we took three steps: to verify participants were on the email list, we asked them to enter their university email address; each survey link could only be used once; and we asked students to describe their parents’ educational background in a survey question. All participants passed these checks. To achieve 80% statistical power with an estimated effect size of *d* = .40 (Bauer et al., 2021), we aimed for at least 156 participants.

Overall, 214 students participated. Most were women (145 women, 63 men, 6 other), of diverse race-ethnicities (67 Latinx, 55 Asian, 40 Black, 33 White, 14 mixed, 6 other), with a relatively low family-household income (income bracket, *Mdn* = $25,000fd–$49,999), *M*_age_ = 18.99, *SD* = .77 years.

#### Procedure

Participants were randomly assigned to complete either the identity-reframing or control material before outcome and demographic measures. Both sets of experimental materials were presented as created by researchers and administrative staff at students’ university. Materials were thus presented as reflecting the university’s views.

#### The Low-SES-Identity-Reframing Intervention

The identity-reframing materials were developed following a design process established in previous research (Bauer & Walton, 2023). This included four semi-structured interviews with low-SES students (three first-generation and low-income students; one first-generation and non–low-income student) from the university with whom we collaborated in Study 2. These interviews helped us better understand and represent low-SES students’ challenges and strengths. Some content that emerged was similar to content in the refugee-reframing intervention; for instance, in both populations, students reported having learned to persevere in dealing with challenges (Bauer et al., 2021). An important difference was that the challenges low-SES students described involved less acute experiences of violence and more chronic experiences of deprivation of capital. We also conducted a pilot study, providing low-SES students with draft intervention materials and checking if participants’ open responses in the reflection exercises to see if the intervention material resonated with them. Since it did, we made no major changes.

Similar to other “wise” social-psychological interventions (Walton & Cohen, 2011; Walton et al., 2023), the low-SES-identity-reframing intervention included three parts: a brief introduction, stories from prior students, and interactive writing exercises. The exercise took students about 11 min to complete, *Mdn* = 11.48, *SD* = 19.83.

The introduction read:

In previous interviews and surveys, many students said that their experiences as first-generation-students were often difficult. But interestingly, many also said that they have learned a lot of useful things as first-generation-students.

Next, four stories illustrated this idea with concrete examples of how individual students developed strengths in facing low-SES–based challenges and used these strengths to succeed in school. One read:

My parents … couldn’t really help me with schoolwork, money or advice on how to succeed in college. I feel like I had to work extra hard compared to many other students. … I had to learn to reach out to others … when I needed help … I also had a part-time job at a library to get a little more money. This was all pretty difficult sometimes. At the same, I feel like it was a good preparation for college. When things get difficult with my schoolwork at [university name], I don’t give up that easily. … Looking back at my experiences makes me feel kinda … well, yeah, proud: I’m where I am because of my hard work, not because of my parents’ money or anything like that. My family and I had it more difficult than many others, but I’ve still been doing reasonably well …—I think I can be proud of that.

As this quotation highlights, the identity-reframing materials addressed both sociocultural (limited parental “advice on how to succeed”) and economic (limited “money”) challenges.

Writing exercises then invited students to share their own strengths rooted in their SES-identity: What “have [you] learned through your experiences as a first-generation student” and how do “you think will you use these strengths to help you succeed at [university name]?”

#### Control Condition

The control condition used a similar format, including stories from prior students and writing exercises illustrating how previous experiences could help students succeed academically. However, these materials focused on study strategies (Kizilcec et al., 2017; Walton et al., 2015) and did not address students’ SES identity.

#### Measures

##### Open-Ended Descriptions of Low-SES–Based Challenges and Strengths

To assess students’ representation of the challenges and strengths related to their identity, we asked students in both conditions, “How do you think being a first-generation student affects your experiences at college (if at all)? Please describe in three to four sentences.” Two research assistants coded the number of challenges (intra-class correlation coefficient [ICC] = .92) and strengths (ICC = .96) students described, as well as the different types of challenges and strengths they described (see section “Results” and Supplemental Material). We used a master-coder approach: One research assistant coded all 214 responses and another coded one third of the data (77 responses) to determine inter-rater reliability. Only codings from the first research assistant were used in analyses.

##### Self-Ratings of Students’ SES-Identity as a Source of Challenge and Academic Success

Complementing open-ended responses, we assessed to what extent students saw their first-generation identity as a resource (“My background as a first-generation student has helped me succeed at [university name] in the past” and “. . . will help me succeed at [university name] in the future”; 1 = *strongly disagree*, 6 = *strongly agree*, α = .78).

We also assessed the extent to which students saw their SES-identity as making academic success more challenging (“My background as a first-generation student has made it more difficult for me to succeed at [university name] in the past,” and “. . . will make it more difficult for me to succeed at [university name] in the future,” 1 = *strongly disagree*, 6 = *strongly agree*, α = .81).

##### Performance on an Academic Task

Following previous research (Apfelbaum et al., 2016; Brannon et al., 2015; Stephens et al., 2012), we asked students to solve 12 verbal anagrams, re-arranging letters of a word (e.g., “cone”) to create a different word (e.g., “once”). Because our goal was to assess whether the treatment would enhance performance on a task linked to students’ identity, we described the task in this way, following previous research (Apfelbaum et al., 2016):

Students have very different backgrounds [which can] provide people with insights for how to think about solving problems … Word puzzles that can help us understand your problem-solving-style.

Importantly, this representation does not specify whether students’ low-SES-identity may improve or impair students’ performance.

To incentivize engagement, students were told that the person with the highest score would win a $100 online shopping voucher. There was no time limit. The primary outcome was the total number of puzzles solved correctly, which reflects both persistence and accuracy. We also assessed accuracy, the ratio of correctly completed and attempted puzzles. Following previous research (Apfelbaum et al., 2016), we considered any typed response an attempt signaling persistence.

### Results

#### Most Common Strengths and Challenges

Table 1 and Supplemental Table S1 show the most commonly mentioned SES-based strengths and challenges.

**Table 1:** The Most Common SES-Based Strengths First-Generation Students Mentioned in Open-Ended Responses (Experiment 1)

Motivation to Work Hard (14%) “I think [being a first-generation student] makes me want to work harder because I know that my parents didn’t have this opportunity.”
Being Able to Deal with Challenges (9%) “Being first generation has given me strength. I know I must work harder to achieve a similar goal … This builds much character.”
Being Able to Relate to Others (6%) “[Being a first-generation student] makes it easier for me to approach professors and teachers. I know how to look for help when I need it.”
Gratitude (6%) “[Being a first-generation student] allows me to not take many of the things that are given to me for granted. I value anything this university is able to give me from the food to the opportunities.”
Pride (4%) “I think [being a first-generation student] comes with a certain level of pride that other students may not get to experience. I know that what I am doing is difficult, and I feel proud to have been able to succeed.”
Having a Different Perspective (2%) “I think [being a first-generation student] gives me a unique perspective and makes me realize the amount of privilege many students here were given to get the opportunities they have now.”

*Note*. Percentages represent the proportion of all responses referencing the respective theme across conditions.

#### Frequency and Ratings of Low-SES–Based Strengths and Challenges

The identity-reframing exercise led first-generation students to recognize more strengths in their low-SES-identity (see Table 2). In open-ended responses, it doubled the percentage of students who spontaneously recognized at least one low-SES–based strength in themselves, from 19% to 42%, χ^2^(1, *N* = 214) = 12.55, odd ratio (*OR*) = 3.08 95% confidence interval [CI] = [1.68, 5.79], *p <* .001. Similarly, students rated their low-SES-identity as a resource for academic success more in the identity-reframing than the control condition (see Table 2).

**Table 2:** Extent to Which Students Recognized Low-SES–Based Strengths and Challenges as Assessed in Open-Ended Responses and Scale Ratings (Experiment 1)

	Low-SES–based strengths (e.g., being motivated)		Low-SES–based challenges (e.g., receiving limited support)	
	Open response: number of strengths mentioned	Scale-rating: SES as resource for success	Open response: number of challenges mentioned	Scale-rating: SES as source of challenge
Control: *M* (*SD*)	0.23 (0.52)	3.69 (1.06)	2.36 (2.07)	3.76 (1.17)
Interv.: *M* (*SD*)	0.85 (1.23)	4.12 (1.01)	2.08 (1.32)	3.75 (1.14)
*p*	<.001	.003	.138	.838
*d*	0.66	0.42	−0.20	−0.03
*d*: 95% CI	[0.39, 0.94]	[0.15, 0.67]	[0.07, −0.47]	[0.24, −0.30]

*Note*. In coding open responses, we captured the number of strengths and the number of challenges students spontaneously mentioned in describing how being a first-generation student has affected their college experience. Scale ratings are on a 1 to 6 scale.

In line with theory (Bauer & Walton, 2023), the identity-reframing did not reduce the extent to which students recognized challenges to academic success based on their low-SES-identity (see Table 2): Participants mentioned an average of roughly two low-SES–based challenges, such as having limited support and resources in open responses, *M* = 2.22, *SE* = 1.41, with no difference between conditions (see Supplemental Table S1). Scale ratings are in line with this finding. Across conditions, students “rather agreed” that their identity makes “it more difficult for me to succeed at (university name)” (see Table 2). This continued recognition of low-SES–based challenges may be important for students to be able to deal with challenges effectively (Chen et al., 2017).

#### Performance on the Academic Task

**Figure 1:**
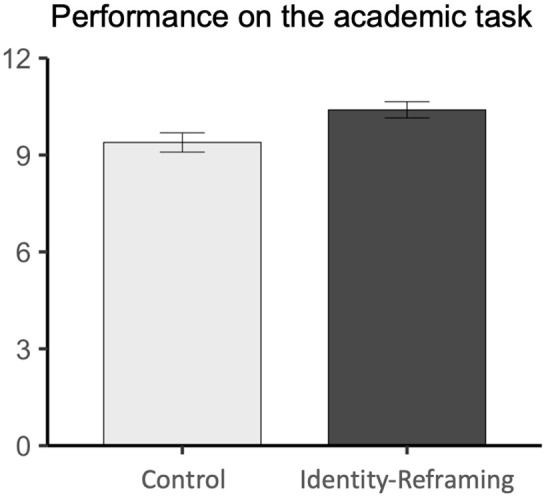
First-Generation Students’ Performance on an Anagram Task (Experiment 1)

As predicted, first-generation students solved 13% more anagrams correctly in the identity-reframing condition (*M* = 10.63, *SD* = 2.51) than in the control condition (*M* = 9.46, *SD* = 3.44), *F*(1, 212) = 7.89, *d* = .38, 95% CI = [.12, .66], *p* = .005 (see Figure 1).

This boost in performance seemed to reflect primarily an increase in persistence: Students attempted more anagrams in the intervention (*M* = 11.15, *SD* = 2.05) than in the control condition (*M* = 10.08, *SD* = 3.30), *F*(1, 212) = 8.05, *d* = .39, 95% CI = [.121, .66], *p* = .005. The accuracy with which they correctly solved attempted problems was high and did not differ by condition (treatment: *M* = 94.47%, *SD* = 2.51; control: *M* = 94.07%, *SD* = 3.44), *F*(1, 212) = .06, *d* = .03, 95% CI = [−.23, .30], *p* = .815.

## Experiment 2: Longitudinal Effects on Achievement

Targeted interventions can propel effects forward in time by triggering self-reinforcing processes. For example, boosts in persistence can help students succeed (see Experiment 1), which, in turn, may reinforce confidence and persistence in a virtuous spiral (e.g., Cohen et al., 2009). To test the potential for identity-reframing to improve achievement over time, we partnered with an online learning platform that provided online coursework to universities. We conducted a pre-registered randomized-controlled experiment in the online portion of a biology class at a U.S. university, tracking 786 students over a semester. Expanding our SES definition, we considered students who reported being first-generation or low-income (operationalized as receiving federal financial aid, Stephens et al., 2014) as low-SES students, randomizing these students to conditions (*N* = 470).

We also obtained data from 316 high-SES students. Since intervention materials specifically addressed low-SES identities, we did not expose high-SES students to the randomized materials (following Bauer et al., 2021). High-SES students were included in the design only on a secondary basis, to explore SES gaps in students’ confidence and grades.

### Method

Additional methodological details are reported in the Supplemental Material.

#### Pre-registration and Statistical Power

Pre-registered hypotheses in Experiment 2 were effects of the identity-reframing intervention on low-SES students’ end-of-semester grades and students’ confidence, assessed roughly 3 weeks post-intervention.

Field studies with long-term behavioral outcomes have different effect size benchmarks than laboratory studies. In education, medium reform effect sizes are considered around 0.10 ≤*d*≤ .20 (Kraft, 2020). Targeted online interventions can achieve similar effects on academic performance, 0.10 ≤*d*≤ .25 (Bauer et al., 2021; Yeager et al., 2016, 2019). With a target of 80% statistical power and an estimated effect size of *d* = .25, we aimed to recruit at least 398 low-SES students.

#### Participants

Participants were 470 low-SES students (68 were first-generation and not low-income; 260 continuing-generation and low-income; 140 first-generation and low-income; 2 low-income with missing first-generation status) and 316 high-SES students (continuing-generation and not low-income).

There were 476 women, 290 men, and 15 non-binary individuals (5 missing). Participants were racially diverse (313 White, 147 Latinx, 130 mixed, 107 Black, 72 Asian, 13 other, 4 missing). Most participants (63%) were in their first university year. Participants majored or intended to major in a variety of fields, most related to biology (top three: 23% biology, 18% health sciences, 17% computer science).

Twelve participants dropped out of the course. We did not receive any performance data for these students, leaving a sample of 774 students for grade analyses. In line with the pre-registration to not exclude participants, we retained these participants for analyses of self-reported confidence.

#### Context and Grades

Our study was conducted in an introduction-to-biology course, which combined in-person instruction with the online learning program in which our intervention was embedded. The online learning program covered 9 weeks of course work over 12 weeks, all of which had to be completed on the online platform. We used the online platform both to implement our materials and to track students’ grades within the learning program. We did not have access to information beyond the platform.

Each week, students received a grade based on their completion of assigned material (50%) and their performance in a test (50%). Students could improve their test score and thus their weekly grade by up to 10% by completing additional assignments (see Supplemental Material for details). If students worked on no material (no learning material, test, or extra assignments) in a week, they received a *0* for that week.

In addition to overall grades, we examined four components of students’ grades:

- the number of weeks students did not work on any material (overall week-grade being *0*),

and, for each week, they did at least some work:

- the percentage of the learning material they completed,
- their test-performance percent score, and
- the number of extra assignments for all weeks in which such assignments could be used to improve scores (i.e., when a student’s weekly performance score was below 100).

We obtained all data from the platform providers.

#### Procedure

There were three sets of materials: a demographic survey (all participants), experimental materials (only low-SES students), and a survey assessing students’ confidence in their potential to succeed (all participants).

The demographic survey and randomized materials were made available together at the end of the first course week and remained open until the fifth course week. Most participants (83%) completed the materials between Weeks 2 and 4. The academic confidence survey was made available beginning in the third week and remained open until the end of the semester. It could be started only after the previous material had been completed. Overall, 76% of participants completed this survey and 91% of those students did so in the third to fifth week, an average 21.97 days after the intervention survey, *SD* = 14.52.

Students who indicated they were a first-generation or low-income student in the demographic survey were automatically randomized to experimental material. All others (i.e., high-SES students) did not receive any experimental material.

#### Experimental Materials

Experimental materials were nearly identical to those in Experiment 1. There were minor changes to fit the context of the university, such as changing the university name and referring to “first-generation/low-income students.”

#### Assessment of Students’ Confidence in their Potential to Succeed in University

We assessed students’ confidence on a percentile scale: “how much potential do you have to be successful at [university name] in comparison to other students?” (*“10%–more potential than 10% of the students and less than 90% of the students”, to “90%”*; Brady et al., 2020; Walton & Cohen, 2007).

### Results

Exploratory analyses showed that the three low-SES groups did not differentially respond to condition on any outcome, group × condition interactions, all *p*s *>* .10, with no consistent trends. We hence report main effects of the intervention among all low-SES students, following pre-registration.

#### Success of Random Assignment

Confirming successful randomization, there was no difference in students’ average grades or any grade-component among low-SES students by condition before participants completed the randomized material, all *p*s > .10.

#### Students’ Confidence in Their Potential to Succeed in University (~3 Weeks Post-Treatment)

Pre-registered treatment comparisons found that low-SES students reported marginally greater confidence in the identity-reframing condition (*M* = 63.95, *SD* = 19.80) than in the control condition (*M* = 60.31, *SD* = 18.73), *F*(1, 355) = 3.140, *d* = .19, 95% CI = [−.02, .40], *p* = .077. This gain rendered an SES gap in confidence—that is, a gap between high-SES students (*M* = 65.74, *SD* = 17.30) and low-SES students in the control condition, *F*(1, 397) = 8.86, *d* = .30, 95% CI = [.10, .50], *p* = .003—non-significant (low-SES intervention group: *M* = 6.42, *SD* = 1.96), *F*(1, 430) = 1.01, *d* = .10, 95% CI = [−.09, .29], *p* = .317.

#### Grades

**Figure 2:**
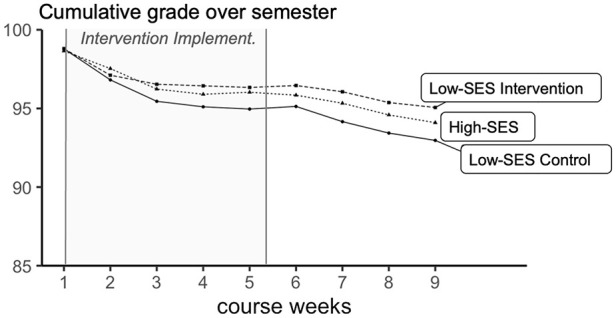
Students’ Cumulative Grades in the Online Learning Program Over One Semester (Experiment 2) *Note*. Intervention Implement. = period of intervention implementation.

As predicted and pre-registered, the intervention boosted students’ final grade in the online learning program (see Table 3 and Figure 2): Over the semester, low-SES students randomized to reflect on the strengths low-SES students develop in contending with low-SES–based challenges earned grades over two percentage-point higher than low-SES students randomized to the active control condition.

**Table 3:** Intervention Effects on Students’ Final Grade and Grade Components in the Online Learning Program (Experiment 2)

	Descriptive: *M (SD)*			Control vs. Int.		Control vs. high SES		Int. vs. high SES	
	Low SES Cont.	Low SES Int.	High SES	*p*	*d*	*p*	*d*	*p*	*d*
Final grade in the learning platform	92.99 (11.73)	95.15 (8.15)	94.12 (9.87)	.020	.22	.237	.11	.182	.11
Weeks no material started^a^	0.49 (1.16)	0.41 (0.87)	0.49 (1.01)	.367	.08	.946	<.01	.348	.08
% Weekly learning material completed^a^	99.44 (1.80)	99.40 (1.92)	99.31 (2.23)	.806	.02	.494	.06	.640	.07
Performance on weekly tests	90.29 (6.72)	92.21 (5.51)	91.80 (5.70)	.001	.32	.006	.25	.389	.04
Extra assignments completed per week	2.45 (2.75)	3.21 (4.03)	2.78 (3.35)	.020	.22	.237	.11	.165	.28

*Note*. Cont. = low-SES control group; Int. = low-SES intervention group.

a Patterns for these variables are consistent with a ceiling effect.

Exploratory analyses examined the four components of grades. Two components showed ceiling effects, with little room for improvement. In an average week, almost all students (95%) started work on the assignments and, once started, students completed all of the learning material 99% of the time. In line with this limited variance, there were no SES gaps or intervention effects on these grade components.

The two remaining components—students’ test-performance and completion of extra-credit assignments—showed more variability. Identity-reframing improved both these outcomes (see Table 3). It increased the number of extra assignments low-SES students completed by 31%, and raised their weekly test performance by two percentage-points, similar to the overall improvement in grades.

Secondary analyses exploring SES gaps (see Table 3) are in line with the idea that identity-reframing may be most effective in improving low-SES students’ performance when there is a baseline SES gap. Identity-reframing effects were significant for the two grade components—number of completed extra credit assignments and test performance—in which control low-SES students descriptively underperformed compared to high-SES students, although this baseline SES gap reached significance only for test performance.

#### Additional Analyses

We conducted a series of additional analyses to better understand the results. First, we examined only performances after students had completed the randomized material. These yielded similar improvements in average grades, weekly test performance, and completed extra assignments, *F*(1, 460), *p* = .025, *d* = .21, 95% CI = [.03, .39], *F*(1, 460), *p* = .005, *d* = .27, 95% CI = [.08, .45], and *F*(1, 460), *p* = .026, *d* = .21, 95% CI = [.03, .39], respectively (see Supplemental Material, Table S11 for details).

**Figure 3:**
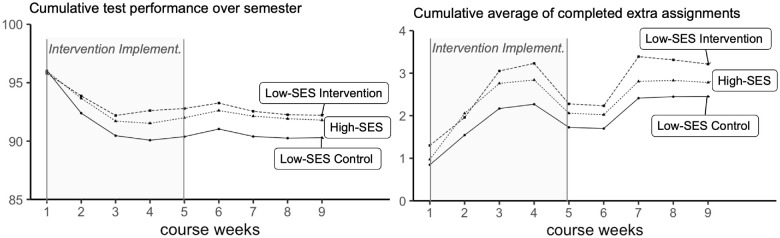
Cumulative Test Performance (Left) and Average Completed Extra Assignments (Right) Over the Semester (Experiment 2) *Note*. Intervention Implement. = time of intervention implementation.

Second, we examined treatment effects on academic performance on a week-by-week basis. As reported in the Supplemental Material (see Tables S2–S10), these analyses show that treatment effects began to emerge shortly after the intervention was completed, in Weeks 2 and 3 (only two participants completed experimental material before completing the Week 1 coursework), and then persisted over the term. Descriptively, from Week 2 to Week 9, students randomized to the identity-reframing condition outperformed students in the control condition in 22 of 24 comparisons (3 outcomes × 8 weeks). While these comparisons were not all significant, this consistent pattern means that cumulative effect sizes grew over time (see Figures 2 and 3 and Supplemental Table S10).

Third, given that there was variability in when students accessed experimental materials, we tested whether this moderated intervention effects. Consistent with past research suggesting that interventions can have larger effects when implemented earlier in a course or school transition (Walton & Wilson, 2018; e.g., Canning et al., 2018), we found larger gains in grades among students who completed the low-SES-reframing exercise earlier (see Supplemental Figure S1).

Fourth, we tested whether the (marginally significant) initial increase in students’ confidence could have contributed to long-term improvement in grades. Exploratory mediation analyses provide some support for this idea with a marginal condition confidence final-grade mediation, 90% CI = [.12, .45].

## General Discussion

Students from low-SES backgrounds are often portrayed as deficient, lacking in academic skills and potential. In the present research, we test the power of inverting this narrative. We represented low-SES students as strong and resourceful specifically by highlighting the strengths students show and develop in contending with the low-SES–based challenges they experience. In two randomized-controlled experiments, one a pre-registered longitudinal field experiment, we show that this representation helped low-SES students recognize strengths in their low-SES-identity and raised their academic performance, including their grades over a semester.

In Experiment 1, we asked first-generation college students how their SES background affected their college experience. Reflecting common deficit narratives, 81% of students in the control group recognized no strengths connected to their SES-identity at all, only challenges. The low-SES-identity-reframing intervention boosted the percentage of students who recognized at least one strength from 19% to 42%. Furthermore, with this reframing, students rated their SES-identity more as a resource that could help them succeed. And then, it did. In Experiment 1, first-generation students who got the identity-reframing exercise persisted more and performed better on an academic task. In Experiment 2, where we implemented the exercise in the online learning program of a biology course, it raised grades among first-generation and low-income students over the semester. This improvement was driven by greater performance on weekly tests, eliminating an SES performance gap. It also arose in greater take up of extra credit assignments, suggesting greater engagement in course material. Week-by-week analyses suggest that intervention-effects emerged quickly and then persisted. Students who participated in the identity-reframing intervention descriptively outperformed students in the control condition in most weeks on most grade components (in 22 of 24 comparisons). Although weekly effects were not always significant, effects on cumulative grades tended to steadily grow over time.

These results show that the widespread narrative that bemoans low-SES students as simply lacking in skills can have self-fulfilling consequences. This narrative limits the extent to which students can access and apply their strengths. Yet, this harm is not inevitable. Even a brief exercise, but one that convincingly represented students as showing strengths in the face of low-SES–based challenges, helped students succeed in their coursework.

Two nuances of this approach merit discussion. First, we represented this strength-based view of low-SES students as the view of students’ university. We did so based on research suggesting that individuals’ performance is shaped not only by individuals’ self-beliefs but also by how they believe they are seen by others (Spencer et al., 2016). It is unclear to what extent intervention-effects are driven by students’ exposure to the intervention-message itself, or also by the experience of being recognized as strong and agentic by their school. Future research could test this by varying the source of the intervention message.

Second, the identity-reframing exercise represented both challenges and strengths; it specifically framed contending with challenges as a source of and evidence for strengths (Bauer & Walton, 2024). It is possible that this aspect of the exercise facilitates an integrated understanding that allows students to fully recognize and contend with low-SES–based challenges while maintaining a positive view of their identity. Indeed, Experiment 1 showed that identity-reframing did not lead low-SES students to downplay SES-based challenges even as they recognized more strengths. By contrast, other strength-based interventions (a) take a broader approach, including to represent strengths such as family and community resources that may not arise from contending with challenges and (b) do not address low-SES–based challenges directly, only strengths (e.g., Hernandez et al., 2021). A risk of narratives that recognize only strengths is that they may be disconfirmed when low-SES students face low-SES–based challenges (see Crum et al., 2023). In contrast, an integrated understanding may help students carry the narrative forward, and use it to contend with challenges over time. Future research could test this idea by directly comparing the identity-reframing exercise with alternatives focused only on strengths.

Future research may also expand on the single-item measure of students’ confidence in their potential, as multiple items can enhance reliability and hence sensitivity to condition effects.

As with all psychological interventions, identity-reframing exercises do not act alone. The processes they trigger can persist and improve long-term outcomes only if circumstances permit (Walton & Yeager, 2020). Identity-reframing may help students make better use of existing resources (e.g., adequate study material, and support). If resources are lacking, the intervention will not substitute. Similarly, the benefits may short-circuit if the school-context contradicts the intervention message, such as if low-SES students are treated as deficient.

Thus, an exciting direction is to develop exercises to reframe low-SES identities for teachers and peers and to test whether such exercises could enhance the effects of identity-reframing interventions with low-SES students. Already research has found that representing low-SES students as having important strengths can help teachers acknowledge these strengths (Belmi et al., 2023; Silverman, Hernandez, & Destin, 2023). Could such improvements in teachers’ views further enhance the benefits of student-level identity-reframing interventions over time?

Such work at multiple levels would move toward creating a culture in which low-SES students are fully recognized as bringing strengths from their SES background to school. These psychosocial supports could then allow capable students to realize their strengths.

## Supplemental Material

sj-docx-1-spp-10.1177_19485506241284806 – Supplemental material for The Strengths of People in Low-SES Positions: An Identity-Reframing Intervention Improves Low-SES Students’ Achievement Over One Semester Supplemental material, sj-docx-1-spp-10.1177_19485506241284806 for The Strengths of People in Low-SES Positions: An Identity-Reframing Intervention Improves Low-SES Students’ Achievement Over One Semester by Christina A. Bauer, Gregory Walton, Veronika Job and Nicole Stephens in Social Psychological and Personality Science

## References

[bibr1-19485506241284806] American Psychological Association. (n.d.). Socioeconomic status. https://www.apa.org/topics/socioeconomic-status

[bibr2-19485506241284806] Apfelbaum E. P. Stephens N. M. Reagans R. E. (2016). Beyond one-size-fits-all: Tailoring diversity approaches to the representation of social groups. Journal of Personality and Social Psychology, 111(4), 547. 27428047 10.1037/pspi0000071

[bibr3-19485506241284806] Avery C. Dynarski S. Turner S. (2020). Low-income students lose ground. Science, 370(6521), 1141. 10.1126/science.abf9375 33273075

[bibr4-19485506241284806] Bauer C. A. Boemelburg R. Walton G. M. (2021). Resourceful actors, not weak victims: Reframing refugees’ stigmatized identity enhances long-term academic engagement. Psychological Science, 32(12), Article 12. 10.1177/09567976211028978 34793270

[bibr5-19485506241284806] Bauer C. A. Job V. Hannover B. (2023). Who gets to see themselves as talented? Biased self-concepts contribute to first-generation students’ disadvantage in talent-focused environments. Journal of Experimental Social Psychology, 108(104501), 1–14. 10.1016/j.jesp.2023.104501

[bibr6-19485506241284806] Bauer C. A. Walton G. (2023). Identity-reframing interventions: How to effectively highlight individuals’ background-specific strengths. Social and Personality Psychology Compass, 18, Article e12830. 10.1111/spc3.12830

[bibr7-19485506241284806] Bauer C. A. Walton G. M. (2024). Liberal paternalism: Weak-victim narratives are common, especially among liberals, and facilitate the disempowering treatment of low-status group members [Manuscript under review].

[bibr8-19485506241284806] Belmi P. Raz K. Neale M. Thomas-Hunt M. (2023). The consequences of revealing first-generational status. Organization Science, 35, 667–697. 10.1287/orsc.2023.1682

[bibr9-19485506241284806] Bourdieu P. (1984). Distinction a social critique of the judgement of taste. In Grusky D.B. Szelényi S. (Eds.), Inequality (pp. 287–318). Routledge.

[bibr10-19485506241284806] Brady S. T. Cohen G. L. Jarvis S. N. Walton G. M. (2020). A brief social-belonging intervention in college improves adult outcomes for black Americans. Science Advances, 6(18), Article 18. 10.1126/sciadv.aay3689 PMC7190359 32426471

[bibr11-19485506241284806] Brannon T. N. Markus H. R. Taylor V. J. (2015). “Two souls, two thoughts,” two self-schemas: Double consciousness can have positive academic consequences for African Americans. Journal of Personality and Social Psychology, 108(4), Article 4. 10.1037/a0038992 25844575

[bibr12-19485506241284806] Canning E. A. Harackiewicz J. M. Priniski S. J. Hecht C. A. Tibbetts Y. Hyde J. S. (2018). Improving performance and retention in introductory biology with a utility-value intervention. Journal of Educational Psychology, 110(6), 834–849. 30294006 10.1037/edu0000244 PMC6168083

[bibr13-19485506241284806] Chen P. Chavez O. Ong D. C. Gunderson B. (2017). Strategic resource use for learning: A self-administered intervention that guides self-reflection on effective resource use enhances academic performance. Psychological Science, 28(6), 774–785. 10.1177/0956797617696456 28447894

[bibr14-19485506241284806] Cohen G. L. Garcia J. Purdie-Vaughns V. Apfel N. Brzustoski P. (2009). Recursive processes in self-affirmation: Intervening to close the minority achievement gap. Science, 324(5925), 400–403. 10.1126/science.1170769 19372432

[bibr15-19485506241284806] Crum A. J. Santoro E. Handley-Miner I. Smith E. N. Evans K. Moraveji N. Achor S. Salovey P. (2023). Evaluation of the “rethink stress” mindset intervention: A metacognitive approach to changing mindsets. Journal of Experimental Psychology: General, 152(9), 2603. 37199967 10.1037/xge0001396

[bibr16-19485506241284806] Durante F. Fiske S. T. (2017). How social-class stereotypes maintain inequality. Current Opinion in Psychology, 18, 43–48. 29221511 10.1016/j.copsyc.2017.07.033 PMC6020691

[bibr17-19485506241284806] Frankenhuis W. E. Nettle D. (2020). The strengths of people in poverty. Current Directions in Psychological Science, 29(1), 16–21. 10.1177/0963721419881154

[bibr18-19485506241284806] Goudeau S. Stephens N. M. Markus H. R. Darnon C. Croizet J.-C. Cimpian A. (2024). What causes social class disparities in education? The role of the mismatches between academic contexts and working-class socialization contexts and how the effects of these mismatches are explained. Psychological Review. Advance online publication. https://www.researchgate.net/profile/Andrei-Cimpian/publication/377111119_What_Causes_Social_Class_Disparities_in_Education_The_Role_of_the_Mismatches_between_Academic_Contexts_and_Working-Class_Socialization_Contexts_and_How_the_Effects_of_These_Mismatches_Are_Explained/links/6595b7a46f6e450f19c89a31/What-Causes-Social-Class-Disparities-in-Education-The-Role-of-the-Mismatches-between-Academic-Contexts-and-Working-Class-Socialization-Contexts-and-How-the-Effects-of-These-Mismatches-Are-Explained.pdf 10.1037/rev0000473 39023935

[bibr19-19485506241284806] Hernandez I. A. Silverman D. M. Destin M. (2021). From deficit to benefit: Highlighting lower-SES students’ background-specific strengths reinforces their academic persistence. Journal of Experimental Social Psychology, 92(104080), Article 104080. 10.1016/j.jesp.2020.104080

[bibr20-19485506241284806] Kizilcec R. F. Saltarelli A. J. Reich J. Cohen G. L. (2017). Closing global achievement gaps in MOOCs. Science, 355(6322), 251–252. 10.1126/science.aag2063 28104856

[bibr21-19485506241284806] Kraft M. A. (2020). Interpreting effect sizes of education interventions. Educational Researcher, 49(4), 241–253. 10.3102/0013189X20912798

[bibr22-19485506241284806] Kraus M. W. Côté S. Keltner D. (2010). Social class, contextualism, and empathic accuracy. Psychological Science, 21(11), 1716–1723. 10.1177/0956797610387613 20974714

[bibr23-19485506241284806] Kray L. J. Galinsky A. D. Thompson L. (2002). Reversing the gender gap in negotiations: An exploration of stereotype regeneration. Organizational behavior and human decision processes, 87(2), 386–409. 10.1006/obhd.2001.2979

[bibr24-19485506241284806] McKay J. Devlin M. (2016). “Low income doesn’t mean stupid and destined for failure”: Challenging the deficit discourse around students from low SES backgrounds in higher education. International Journal of Inclusive Education, 20(4), 347–363.

[bibr25-19485506241284806] Mehta S. S. Newbold J. J. O’Rourke M. A. (2011). Why do first-generation students fail? College Student Journal, 45(1), Article 1.

[bibr26-19485506241284806] Riggs L. (2014, December 31). First-generation college-goers: Unprepared and behind. The Atlantic. https://www.theatlantic.com/education/archive/2014/12/the-added-pressure-faced-by-first-generation-students/384139/

[bibr27-19485506241284806] Silverman D. M. Hernandez I. A. Destin M. (2023). Educators’ beliefs about students’ socioeconomic backgrounds as a pathway for supporting motivation. Personality and Social Psychology Bulletin, 49(2), 215–232. 34964382 10.1177/01461672211061945

[bibr28-19485506241284806] Spencer S. J. Logel C. Davies P. G. (2016). Stereotype threat. Annual Review of Psychology, 67, 415–437. 10.1146/annurev-psych-073115-103235 26361054

[bibr29-19485506241284806] Stephens N. M. Fryberg S. A. Markus H. R. Johnson C. S. Covarrubias R. (2012). Unseen disadvantage: How American universities’ focus on independence undermines the academic performance of first-generation college students. Journal of Personality and Social Psychology, 102(6), Article 6. 10.1037/a0027143 22390227

[bibr30-19485506241284806] Stephens N. M. Hamedani M. G. Destin M. (2014). Closing the social-class achievement gap: A difference-education intervention improves first-generation students’ academic performance and all students’ college transition. Psychological Science, 25(4), Article 4. 10.1177/0956797613518349 24553359

[bibr31-19485506241284806] Walton G. M. Cohen G. L. (2007). A question of belonging: Race, social fit, and achievement. Journal of Personality and Social Psychology, 92(1), Article 1. 10.1037/0022-3514.92.1.82 17201544

[bibr32-19485506241284806] Walton G. M. Cohen G. L. (2011). A brief social-belonging intervention improves academic and health outcomes of minority students. Science, 331(6023), Article 6023. 10.1126/science.1198364 21415354

[bibr33-19485506241284806] Walton G. M. Logel C. Peach J. M. Spencer S. J. Zanna M. P. (2015). Two brief interventions to mitigate a “chilly climate” transform women’s experience, relationships, and achievement in engineering. Journal of Educational Psychology, 107(2), 468–485.

[bibr34-19485506241284806] Walton G. M. Murphy M. C. Logel C. Yeager D. S. Goyer J. P. Brady S. T. Krol N. (2023). Where and with whom does a brief social-belonging intervention raise college achievement. Science, 380, 499–505. 37141344 10.1126/science.ade4420

[bibr35-19485506241284806] Walton G. M. Wilson T. D. (2018). Wise interventions: Psychological remedies for social and personal problems. Psychological Review, 125(5), Article 5. 10.1037/rev0000115 30299141

[bibr36-19485506241284806] Walton G. M. Yeager D. S. (2020). Seed and soil: Psychological affordances in contexts help to explain where wise interventions succeed or fail. Current Directions in Psychological Science, 29(3), Article 3. 10.1177/0963721420904453 PMC7384695 32719574

[bibr37-19485506241284806] Yeager D. S. Hanselman P. Walton G. M. Murray J. S. Crosnoe R. Muller C. Tipton E. Schneider B. Hulleman C. S. Hinojosa C. P. (2019). A national experiment reveals where a growth mindset improves achievement. Nature, 573(7774), 364–369. 10.1038/s41586-019-1466-y 31391586 PMC6786290

[bibr38-19485506241284806] Yeager D. S. Walton G. M. Brady S. T. Akcinar E. N. Paunesku D. Keane L. Kamentz D. Ritter G. Duckworth A. L. Urstein R. (2016). Teaching a lay theory before college narrows achievement gaps at scale. Proceedings of the National Academy of Sciences, 113(24), 3341–3348. 10.1073/pnas.1524360113 PMC4914175 27247409

