# Supplementary material for: The Strengths of People in Low-SES Positions: An Identity-Reframing Intervention Improves Low-SES Students’ Achievement Over One Semester
Source: Soc Psychol Personal Sci. 2024 Oct 20;16(1):45–55. doi: 10.1177/19485506241284806 (PMC11585444; doi:10.1177/19485506241284806)
Supplement: sj-docx-1-spp-10.1177_19485506241284806 – Supplemental material for The Strengths of People in Low-SES Positions: An Identity-Reframing Intervention Improves Low-SES Students’ Achievement Over One Semester [file sj-docx-1-spp-10.1177_19485506241284806.docx]

Table of Contents

[1. Experiment 2: Details on Methods and Participants 2](#_Toc173141655)

[2. Experiments 1 and 2: Exploratory analyses on the role of ethnicity 4](#_Toc173141656)

[3. Experiment 1: Item-level analyses of students’ views of their SES-identity 5](#_Toc173141657)

[4. Experiment 1: Table of reported background-specific challenges 6](#_Toc173141658)

[5. Experiment 2: Week-by-week patterns of academic performance 7](#_Toc173141659)

[6. Experiment 2: Intervention Effects Including only Post-Intervention Outcomes 14](#_Toc173141660)

[7. Experiment 2: Exploratory analyses on the role of timing 15](#_Toc173141661)

[8. Experiment 2: Information on Time Spent on the Learning-Platform 17](#_Toc173141662)

[References 18](#_Toc173141663)

# Experiment 2: Details on Methods and Participants

**Participants.** Following previous work (Stephens et al., 2014), we defined low-income students as those who reported that they received federal financial aid such as a Pell Grant. Eligibility for student aid depends on multiple factors including family income, wealth, and size. In our sample, 56% of students indicated receiving aid. Nationwide, in 2020-21, 32% of students did (National Center for Education Statistics, 2023).

**Materials.** There were three sets of materials: i) a brief demographic survey (all participants), ii) the randomized intervention or control materials (low-SES participants), and iii) a survey assessing students’ confidence in their potential to succeed (all participants).

The demographic survey and randomized materials were made available together at the end of the first course week along with informed consent forms and remained open until the fifth course week. Very few (0.4%) participating low-SES students completed these materials before completing the course work for week 1; 46% of low-SES students completed these materials between weeks 1 and 2, 37% between weeks 2 and 3, 6% between weeks 3 and 4, and 10% between weeks 4 and 5.

The academic confidence survey was made available beginning in week 3 and remined open until the end of the semester. It could be started only after the previous material (demographic survey and, for low-SES students only, intervention or control material) had been completed.

While the completion of our study materials was optional, they were implemented as part of the regular learning material and the course instructor encouraged students to complete them. Of the 470 low-SES participants, 357 (76%) completed the confidence survey. Among our 316 higher-SES student participants, 237 (75%) did. Overall, 91% of students who completed the survey did so in the third to fifth week, an average 21.97 days after the intervention survey, *SD*=14.52, *range*= 0-92 days.

**Parallel Study.** As is indicated in the pre-registration, we learned that another research team was conducting another randomized intervention in parallel to our study with the same participants (i.e., with a separate randomization). This intervention was unrelated to students’ identities and had no effects, either on the outcomes examined here or on other outcomes the other research team assessed.

**Grade and Grade Components.** Each week in the online-learning program students studied a different topic (e.g., Photosynthesis) and completed materials and a test for this topic online. The online materials were designed by the class instructor, who also designed the in-class materials. Each week, students received a grade based on their completion of the assigned material (50%) and their test performance (50%). However, students could improve their test score and thus their weekly grade by up to 10% by completing additional assignments (see Supplement for details). If students worked on no material (no learning material, test, or extra assignment) in a week, they received a *0* for that week. The final grade for the learning program was calculated as the mean of the nine weekly grades, with the lowest grade dropped (for week-by-week analyses, we dropped the first occurring lowest score, if there were multiple weeks with the same lowest score value).

The exact formula Realizeit uses is:

*if(if(Progress < 0, 0.0, Progress) * 0.5 + if(Test_performance < 0, 0.0, Test_performance) * 0.5>=99, 100,*

*if(if(Progress < 0, 0.0, Progress) * 0.5 + if(Test_performance < 0, 0.0, Test_performance) * 0.5> if(Progress < 0, 0.0, Progress) * 0.5 + if(Test_performance < 0, 0.0, Test_performance) * 0.4 + If(NumPract+NumRevise>10, 10, NumPract+NumRevise), if(Progress < 0, 0.0, Progress) * 0.5 + if(KS < 0, 0.0, KS) * 0.5, if(Progress < 0, 0.0, Progress) * 0.5 + if(Test_performance < 0, 0.0, Test_performance) * 0.4 + If(NumPract+NumRevise>10, 10, NumPract+NumRevise) ))*

with Progress= progress students made on the learning material and

NumPract = Number of additional exercises students practiced

NumRevise = Number of revisions students worked on

This means:

- Students’ grades are usually calculated by averaging their test performance score with their progress score (both on a scale from 0-100). If this average is at least 99, it is rounded to 100.
- However, students could also improve their test performance score by completing additional assignments. If the following score is higher than the simple average score, this score will count:

*Progress*0.5+Test_performance*0.4 + one point to a maximum of ten for each additional assignments*

# Experiments 1 and 2: Exploratory analyses on the role of ethnicity

In exploratory analyses, we tested whether students’ ethnicity moderated identity-reframing intervention effects. To maximize power, we compared White students to all other students, which we consider as BIPOC students.

In Experiment 1, analyses on the six investigated measures (four measures of students’ SES-identity-views and two performance components) yielded no significant race-ethnicity × condition interactions, all *p*s>.10.

In Experiment 2, analyses on our five outcomes yielded three non-significant interactions (number of completed assignments, % of completed learning-material, no material started), *p*s>.10, a significant interaction on test performance, *F*(1,460)=9.09, *η^2^=.*02, *p*=.003*,* and a marginally significant interaction on self-reported confidence *F*(1,356)=3.04, *η^2^=.*01, *p*=.082. The patterns for the latter two interactions were in opposite directions. The identity-reframing effect on test performance was more pronounced among BIPOC low-SES students, *F*(1, 264)=19.36, *η^2^=.*07, *p*<.001, than among White low-SES students, *F*(1,196)=0.04, *p*=.842. However, the effect on confidence was more marginally pronounced among White low-SES students, *F*(1,768)=9.67, *η^2^=.*05, *p*=.006, than among BIPOC low-SES students, *F*(1,199)=0.05, *p*=.822.

These analyses should be treated with caution. Moderation analyses usually require at least twice the sample size than the simple condition effects which our studies were powered for, leading to an increased risk of both false-positives and false-negatives. The exploratory nature of these tests and the conduct of multiple tests further contribute to this risk. Yet, exploring the role of ethnicity in identity-reframing interventions is an important area for future research.

# Experiment 1: Item-level analyses of students’ views of their SES-identity

In the following, we report item-level analyses of the scales assessing the extent to which students saw their SES-identity as a resource and as a challenge. Both scales contained one item focused on the past and one item focused on the future (e.g., “My background as a first-generation student has helped me succeed at [university name] in the past” and “…will help me succeed at [university name] in the future”). For both scales, results were similar for the past- and the future-oriented item.

**Self-ratings of students’ SES-identity as a resource for academic success.** Both items showed condition effects. Students in the identity-reframing condition, as compared to the control condition, were more likely to endorse that their first-generation identity had helped them succeed at their university in the past (*M*=3.90, *SD*=1.08 vs. *M*=3.46, *SD*=1.18) and that it would help them do so in the future (*M*=4.34, *SD*=1.15 vs. *M*=3.91, *SD*=1.19), *F*(1, 212)=8.06, *d*=.39, 95%CI=[.12, .66], *p*=.005, *F*(1, 212)=7.13, *d*=.37, 95%CI=[.10, .64], *p*=.008, respectively.

**Self-ratings of students’ SES-identity as a source of challenge to academic success.** There was no condition effect on either of the two items assessing the extent to which students saw their SES-background as a source of challenge. Across conditions, students “rather agreed” that their identity “has made” (*M*=3.73, *SD*=1.23 vs. *M*=371, *SD*=1.30) and “will make” (*M*=3.73, *SD*=1.26 vs. *M*=3.82, *SD*=1.22) “it more difficult for me to succeed at [university name],” *F*(1, 212)=.02, *p*=.900, *F*(1, 212)=.27, *p*=.607, respectively.

# Experiment 1: Table of reported background-specific challenges

**Table S1.** Experiment 1: Common SES-based challenges students mentioned in open-ended responses.

| **Having More Limited Support in Academia (53%)** *"Navigating this new world [university] without the support of your parents due to their lack of experiences is difficult. "* |
| --- |
| **Being Excluded in Academia (20%)** *"[I feel] that a huge part of the resources and community isn’t 'meant for me.' ”* |
| **Having Limited Resources (15%)** *"it can be hard to keep up with kids who received expensive, private school education if one did not have the same privilege."* |
| **Well-Being & Mental Health (14%)** *"[Being a first-generation student] also makes me anxious and stressed more than the usual student as I have so many weights on my back besides academics."* |
| **Others' Expectations (12%)** *"I feel like there is a lot of pressure to make my family proud given the hard work they put in for me to be here."* |

*Note.* Percentages represent the proportion of all responses referencing the respective theme (in both the intervention and control condition). Some participants referenced multiple themes and were coded accordingly.

# Experiment 2: Week-by-week patterns of academic performance

On the following pages, we report week-by-week and cumulative effects on students’ academic performance (test performance, completion of extra credit assignments, and overall grades). For each outcome, we report three tables:

- Week-by-week comparisons of low-SES students in the intervention condition, low-SES students in the control condition, and high-SES students (Tables S2, S5, and S8).
- For weeks 1-4, we separately report intervention-vs.-control comparisons for low-SES students who had already completed the randomized intervention material (post-intervention) and low-SES students who had not yet completed these materials (pre-intervention) (Tables S3, S6, and S9).
- Cumulative effects comparing low-SES students in the intervention condition, low-SES students in the control condition, and high-SES students, aggregating effects over weeks (Tables S4, S7, and S10

**Table S2.** Experiment 2: Week-by-week intervention effects on test performance (all participants).

|  | **Low-SES Students,**  **Control** | | **Low-SES Students,**  **Intervention** | | **Low-SES Students,**  **Control vs. Interv.** | | **High-SES**  **Students** | | **Low-SES Control**  **vs. High-SES** | | **Low-SES Interv.**  **vs. High-SES** | |
| --- | --- | --- | --- | --- | --- | --- | --- | --- | --- | --- | --- | --- |
|  | ***M*** | ***SD*** | ***M*** | ***SD*** | ***p*** | ***d*** | ***M*** | ***SD*** | ***p*** | ***d*** | ***p*** | ***d*** |
| **week 1** | 96.04 | 8.30 | 95.79 | 7.73 | 0.742 | - | 95.98 | 6.93 | 0.935 | - | 0.757 | - |
| **week 2** | 89.11 | 13.33 | 92.02 | 11.00 | 0.012 | 0.24 | 91.22 | 11.64 | 0.060 | 0.17 | 0.419 | - |
| **week 3** | 89.31 | 13.54 | 93.92 | 9.80 | <.001 | 0.40 | 90.98 | 12.51 | 0.157 | - | 0.003 | 0.26 |
| **week 4** | 91.75 | 11.39 | 93.57 | 10.35 | 0.076 | 0.17 | 94.10 | 7.92 | 0.006 | 0.25 | 0.501 | - |
| **week 5** | 86.96 | 10.33 | 89.10 | 10.54 | 0.031 | 0.21 | 87.83 | 12.34 | 0.407 | - | 0.200 | - |
| **week 6** | 94.61 | 9.08 | 95.75 | 6.94 | 0.134 | - | 95.97 | 7.18 | 0.063 | 0.17 | 0.721 | - |
| **week 7** | 86.73 | 10.21 | 88.44 | 9.82 | 0.075 | 0.17 | 89.45 | 8.50 | 0.001 | 0.3 | 0.197 | - |
| **week 8** | 89.87 | 11.18 | 90.36 | 11.56 | 0.663 | - | 90.60 | 10.73 | 0.487 | - | 0.818 | - |
| **week 9** | 91.10 | 9.94 | 91.91 | 9.33 | 0.387 | - | 91.26 | 10.70 | 0.868 | - | 0.465 | - |

**Table S3.** Experiment 2: Week-by-week intervention effects on test performance among low-SES students, divided by participants who had already completed intervention material (post-intervention) and participants who had not yet (pre-intervention).

|  | **Post-Intervention** | | | | | | | **Pre-Intervention** | | | | | | |
| --- | --- | --- | --- | --- | --- | --- | --- | --- | --- | --- | --- | --- | --- | --- |
|  | **Control Group** | | **Intervention Group** | | **Comparison** | | | **Control Group** | | **Intervention Group** | | **Comparison** | | |
|  | ***M*** | ***SD*** | ***M*** | ***SD*** | ***N*** | ***p*** | ***d*** | ***M*** | ***SD*** | ***M*** | ***SD*** | ***N*** | ***p*** | ***d*** |
| **week 1** | - | - | 99 | 0 | 2 | - | - | 96.04 | 8.30 | 95.76 | 7.75 | 443 | 0.717 | - |
| **week 2** | 89.70 | 12.30 | 92.37 | 10.75 | 203 | 0.102 | - | 88.72 | 14.02 | 91.66 | 11.29 | 241 | 0.075 | 0.23 |
| **week 3** | 89.99 | 12.97 | 94.24 | 9.39 | 369 | <.001 | 0.38 | 86.91 | 15.29 | 91.31 | 12.61 | 71 | 0.219 | - |
| **week 4** | 91.71 | 11.33 | 93.67 | 10.26 | 404 | 0.069 | 0.18 | 92.00 | 12.04 | 92.24 | 11.69 | 43 | 0.95 | - |

**Table S4.** Experiment 2: Cumulative intervention effects on average test performance (all participants).

|  | **Low-SES Students,**  **Control** | | **Low-SES Students,**  **Intervention** | | **Low-SES Students,**  **Control vs. Interv.** | | **High-SES**  **Students** | | **Low-SES Control**  **vs. High-SES** | | **Low-SES Interv.**  **vs. High-SES** | |
| --- | --- | --- | --- | --- | --- | --- | --- | --- | --- | --- | --- | --- |
|  | ***M*** | ***SD*** | ***M*** | ***SD*** | ***p*** | ***d*** | ***M*** | ***SD*** | ***p*** | ***d*** | ***p*** | ***d*** |
| **week 1** | 96.04 | 8.30 | 95.79 | 7.73 | 0.742 | - | 95.98 | 6.93 | 0.935 | - | 0.757 | - |
| **week 2** | 92.38 | 8.55 | 93.86 | 7.20 | 0.045 | 0.19 | 93.65 | 6.95 | 0.063 | 0.17 | 0.728 | - |
| **week 3** | 91.20 | 8.01 | 93.81 | 6.76 | <.001 | 0.36 | 92.77 | 7.07 | 0.019 | 0.21 | 0.077 | 0.15 |
| **week 4** | 91.29 | 7.66 | 93.78 | 6.14 | <.001 | 0.36 | 93.06 | 6.08 | 0.003 | 0.26 | 0.170 | - |
| **week 5** | 90.38 | 7.12 | 92.78 | 6.07 | <.001 | 0.37 | 91.99 | 6.23 | 0.006 | 0.24 | 0.131 | - |
| **week 6** | 91.04 | 6.73 | 93.25 | 5.59 | <.001 | 0.36 | 92.61 | 5.73 | 0.004 | 0.26 | 0.180 | - |
| **week 7** | 90.40 | 6.69 | 92.55 | 5.58 | <.001 | 0.35 | 92.13 | 5.52 | 0.001 | 0.29 | 0.381 | - |
| **week 8** | 90.25 | 6.72 | 92.25 | 5.64 | 0.001 | 0.33 | 91.92 | 5.66 | 0.002 | 0.27 | 0.482 |  |
| **week 9** | 90.29 | 6.72 | 92.21 | 5.51 | 0.001 | 0.32 | 91.80 | 5.70 | 0.006 | 0.25 | 0.389 | - |

**Table S5.** Experiment 2: Week-by-week intervention effects on weekly average number of completed extra credit assignments (all participants).

|  | **Low-SES Students,**  **Control** | | | **Low-SES Students,**  **Intervention** | | | **Low-SES Students,**  **Control vs. Interv.** | | **High-SES**  **Students** | | | **Low-SES Control**  **vs. High-SES** | | **Low-SES Interv.**  **vs. High-SES** | |
| --- | --- | --- | --- | --- | --- | --- | --- | --- | --- | --- | --- | --- | --- | --- | --- |
|  | ***N*** | ***M*** | ***SD*** | ***N*** | ***M*** | ***SD*** | ***p*** | ***d*** | ***N*** | ***M*** | ***SD*** | ***p*** | ***d*** | ***p*** | ***d*** |
| **week 1** | 39 | 0.85 | 1.42 | 63 | 1.30 | 1.75 | 0.174 | - | 74 | 0.97 | 1.30 | 0.635 | - | 0.210 | - |
| **week 2** | 130 | 1.65 | 2.99 | 147 | 2.18 | 3.32 | 0.160 | - | 180 | 2.37 | 4.01 | 0.084 | - | 0.658 | - |
| **week 3** | 124 | 2.58 | 3.72 | 111 | 4.32 | 5.73 | 0.006 | 0.37 | 159 | 3.04 | 4.41 | 0.356 | - | 0.038 | 0.26 |
| **week 4** | 204 | 0.68 | 1.61 | 243 | 0.70 | 1.56 | 0.856 | - | 299 | 0.78 | 2.02 | 0.558 | - | 0.648 | - |
| **week 5** | 194 | 2.57 | 4.07 | 224 | 3.67 | 6.69 | 0.046 | 0.20 | 278 | 3.05 | 5.95 | 0.330 | - | 0.270 | - |
| **week 6** | 68 | 1.38 | 1.92 | 73 | 1.49 | 2.04 | 0.741 | - | 85 | 1.75 | 2.28 | 0.286 | - | 0.455 | - |
| **week 7** | 192 | 5.44 | 7.75 | 231 | 7.30 | 12.89 | 0.080 | 0.17 | 266 | 6.12 | 9.39 | 0.413 | - | 0.239 | - |
| **week 8** | 141 | 3.13 | 4.71 | 153 | 2.70 | 3.73 | 0.378 | - | 179 | 3.14 | 4.88 | 0.993 | - | 0.363 | - |
| **week 9** | 132 | 2.57 | 3.97 | 151 | 2.09 | 3.00 | 0.253 | - | 180 | 2.48 | 3.57 | 0.833 | - | 0.294 | - |

*Note.* As described in the main text, only students who could improve their grades (i.e., students whose test performance was below 100) were included in these analyses, limiting the power of non-cumulative analyses on some weeks

**Table S6.** Experiment 2: Week-by-week intervention effects on weekly average number of completed extra credit assignments among low-SES students*,* divided by participants who had already completed intervention material (post-intervention) and participants who had not yet (pre-intervention).

|  | **Post-Intervention** | | | | | | | **Pre-Intervention** | | | | | | |
| --- | --- | --- | --- | --- | --- | --- | --- | --- | --- | --- | --- | --- | --- | --- |
|  | **Control Group** | | **Intervention Group** | | **Comparison** | | | **Control Group** | | **Intervention Group** | | **Comparison** | | |
|  | ***M*** | ***SD*** | ***M*** | ***SD*** | ***N*** | ***p*** | ***d*** | ***M*** | ***SD*** | ***M*** | ***SD*** | ***N*** | ***p*** | ***d*** |
| **week 1** | - | - | - | - | 0 | - | - | 0.85 | 1.42 | 1.30 | 1.75 | 102 | 0.174 | - |
| **week 2** | 1.29 | 1.99 | 2.73 | 4.15 | 131 | 0.021 | 0.42 | 1.88 | 3.49 | 1.54 | 1.79 | 146 | 0.469 | - |
| **week 3** | 2.55 | 3.62 | 4.55 | 5.92 | 191 | 0.006 | 0.40 | 2.66 | 4.06 | 2.50 | 3.40 | 44 | 0.906 | - |
| **week 4** | 0.69 | 1.54 | 0.76 | 1.61 | 404 | 0.652 | - | 0.62 | 2.08 | 0.00 | 0.00 | 43 | 0.231 | - |

**Table S7.** Experiment 2: Cumulative intervention effects on weekly average number of completed extra credit assignments (all participants).

|  | **Low-SES Students,**  **Control** | | | **Low-SES Students,**  **Intervention** | | | **Low-SES Students,**  **Control vs. Interv.** | | **High-SES**  **Students** | | | **Low-SES Control**  **vs. High-SES** | | **Low-SES Interv.**  **vs. High-SES** | |
| --- | --- | --- | --- | --- | --- | --- | --- | --- | --- | --- | --- | --- | --- | --- | --- |
|  | ***N*** | ***M*** | ***SD*** | ***N*** | ***M*** | ***SD*** | ***p*** | ***d*** | ***N*** | ***M*** | ***SD*** | ***p*** | ***d*** | ***p*** | ***d*** |
| **week 1** | 39 | 0.85 | 1.42 | 63 | 1.30 | 1.75 | 0.174 | - | 74 | 0.97 | 1.30 | 0.174 | - | 0.210 | - |
| **week 2** | 147 | 1.54 | 2.86 | 169 | 1.96 | 3.05 | 0.216 | - | 205 | 2.05 | 3.74 | 0.216 | - | 0.790 | - |
| **week 3** | 188 | 1.89 | 2.84 | 196 | 2.93 | 4.35 | 0.006 | 0.28 | 254 | 2.46 | 3.74 | 0.081 | 0.17 | 0.220 | - |
| **week 4** | 211 | 1.32 | 2.04 | 150 | 1.62 | 2.49 | 0.159 | 0.13 | 311 | 1.56 | 2.47 | 0.235 | - | 0.780 | - |
| **week 5** | 211 | 1.73 | 2.23 | 251 | 2.28 | 2.98 | 0.027 | 0.21 | 312 | 2.06 | 3.09 | 0.184 | - | 0.391 | - |
| **week 6** | 211 | 1.70 | 2.17 | 251 | 2.23 | 2.87 | 0.027 | 0.21 | 312 | 2.02 | 2.90 | 0.027 | 0.21 | 0.394 | - |
| **week 7** | 211 | 2.41 | 2.79 | 251 | 3.39 | 4.38 | 0.005 | 0.26 | 312 | 2.81 | 3.64 | 0.005 | 0.26 | 0.085 | 0.15 |
| **week 8** | 211 | 2.45 | 2.82 | 251 | 3.31 | 4.16 | 0.011 | 0.24 | 312 | 2.83 | 3.50 | 0.011 | 0.24 | 0.135 | - |
| **week 9** | 211 | 2.45 | 2.75 | 251 | 3.21 | 4.03 | 0.020 | 0.22 | 312 | 2.78 | 3.35 | 0.020 | 0.22 | 0.165 | - |

**Table S8.** Experiment 2: Week-by-week intervention effects on overall grades (all participants).

|  | **Low-SES Students,**  **Control** | | **Low-SES Students,**  **Intervention** | | **Low-SES Students,**  **Control vs. Interv.** | | **High-SES**  **Students** | | **Low-SES Control**  **vs. High-SES** | | **Low-SES Interv.**  **vs. High-SES** | |
| --- | --- | --- | --- | --- | --- | --- | --- | --- | --- | --- | --- | --- |
|  | ***M*** | ***SD*** | ***M*** | ***SD*** | ***p*** | ***d*** | ***M*** | ***SD*** | ***p*** | ***d*** | ***p*** | ***d*** |
| **week 1** | 98.79 | 3.68 | 98.80 | 3.21 | 0.985 | - | 98.65 | 3.35 | 0.665 | - | 0.618 | - |
| **week 2** | 95.17 | 9.35 | 96.08 | 10.33 | 0.357 | - | 96.36 | 7.85 | 0.145 | - | 0.735 | - |
| **week 3** | 94.28 | 13.50 | 96.42 | 11.96 | 0.084 | 0.17 | 95.19 | 12.84 | 0.457 | - | 0.265 | - |
| **week 4** | 94.48 | 14.93 | 95.98 | 13.62 | 0.276 | - | 96.59 | 10.59 | 0.066 | - | 0.562 | - |
| **week 5** | 92.30 | 13.98 | 94.60 | 6.38 | 0.034 | 0.22 | 93.49 | 11.50 | 0.337 | - | 0.213 | - |
| **week 6** | 95.97 | 14.28 | 97.10 | 11.42 | 0.352 | - | 95.20 | 17.94 | 0.607 | - | 0.149 | - |
| **week 7** | 87.55 | 23.14 | 93.01 | 13.03 | 0.005 | 0.30 | 91.44 | 17.07 | 0.047 | 0.20 | 0.289 | - |
| **week 8** | 88.04 | 26.19 | 90.39 | 23.50 | 0.345 | - | 89.16 | 25.30 | 0.651 | - | 0.582 | - |
| **week 9** | 89.24 | 25.47 | 92.50 | 19.46 | 0.136 | - | 90.17 | 24.39 | 0.686 | - | 0.238 | - |

**Table S9.** Experiment 2: Week-by-week intervention effects on overall grades among low-SES students, divided by participants who had already completed intervention material (post-intervention) and participants who had not yet (pre-intervention).

|  | **Post-Intervention** | | | | | | | **Pre-Intervention** | | | | | | |
| --- | --- | --- | --- | --- | --- | --- | --- | --- | --- | --- | --- | --- | --- | --- |
|  | **Control Group** | | **Intervention Group** | | **Comparison** | | | **Control Group** | | **Intervention Group** | | **Comparison** | | |
|  | ***M*** | ***SD*** | ***M*** | ***SD*** | ***N*** | ***p*** | ***d*** | ***M*** | ***SD*** | ***M*** | ***SD*** | ***N*** | ***p*** | ***d*** |
| **week 1** | - | - | 100.00 | 0.00 | 2 | - | - | 98.79 | 3.68 | 98.79 | 3.22 | 412 | 0.989 | - |
| **week 2** | 95.93 | 5.42 | 97.40 | 4.14 | 186 | 0.038 | 0.31 | 94.66 | 11.26 | 94.76 | 13.92 | 221 | 0.951 | - |
| **week 3** | 94.59 | 12.68 | 97.35 | 8.12 | 356 | 0.013 | 0.27 | 95.50 | 6.30 | 92.77 | 19.85 | 65 | 0.425 | - |
| **week 4** | 94.93 | 13.72 | 96.39 | 12.51 | 386 | 0.275 | - | 93.31 | 19.75 | 91.11 | 23.49 | 44 | 0.739 | - |

**Table S10.** Experiment 2: Cumulative intervention effects on overall grades (all participants).

|  | **Low-SES Students,**  **Control** | | **Low-SES Students,**  **Intervention** | | **Low-SES Students,**  **Control vs. Interv.** | | **High-SES**  **Students** | | **Low-SES Control**  **vs. High-SES** | | **Low-SES Interv.**  **vs. High-SES** | |
| --- | --- | --- | --- | --- | --- | --- | --- | --- | --- | --- | --- | --- |
|  | ***M*** | ***SD*** | ***M*** | ***SD*** | ***p*** | ***d*** | ***M*** | ***SD*** | ***p*** | ***d*** | ***p*** | ***d*** |
| **week 1** | 98.79 | 3.68 | 98.80 | 3.21 | 0.985 | - | 98.65 | 3.35 | 0.665 | - | 0.618 | - |
| **week 2** | 96.81 | 7.80 | 97.11 | 9.34 | 0.711 | - | 97.53 | 6.42 | 0.249 | - | 0.528 | - |
| **week 3** | 97.38 | 8.51 | 97.93 | 8.24 | 0.484 | - | 98.06 | 4.76 | 0.245 | - | 0.406 | - |
| **week 4** | 96.60 | 8.46 | 97.38 | 7.65 | 0.298 | - | 97.74 | 4.56 | 0.046 | 0.18 | 0.242 | - |
| **week 5** | 95.56 | 8.92 | 96.90 | 6.37 | 0.062 | 0.18 | 96.57 | 6.34 | 0.131 | - | 0.273 | - |
| **week 6** | 95.13 | 9.08 | 96.45 | 7.03 | 0.077 | 0.17 | 95.84 | 7.21 | 0.318 | - | 0.310 | - |
| **week 7** | 94.16 | 9.93 | 96.06 | 6.51 | 0.014 | 0.23 | 95.33 | 7.45 | 0.125 | - | 0.221 | - |
| **week 8** | 93.43 | 10.97 | 95.37 | 7.68 | 0.026 | 0.21 | 94.59 | 8.88 | 0.186 | - | 0.268 | - |
| **week 9** | 92.99 | 11.73 | 95.15 | 8.15 | 0.02 | 0.22 | 94.12 | 9.87 | 0.237 | - | 0.182 | - |

*Note.* Students could drop their lowest weekly grade. We dropped the first occurrence of their lowest grade.

# Experiment 2: Intervention Effects Including only Post-Intervention Outcomes

**Table S11.** Intervention effects on students’ average grade, test performance, and completion of extra assignments including only post-intervention outcomes (Experiment 2).

|  | **Control Group** | **Intervention Group** | |  | |
| --- | --- | --- | --- | --- | --- |
|  | ***M(SD)*** | ***M(SD)*** | | ***p*** | ***d*** |
| Average Weekly Grade | 92.24 (12.81) | | 94.54 (9.02) | .025 | .21 |
| Performance on Weekly Tests | 89.96 (6.67) | 91.64 (6.02) | | .005 | .27 |
| Extra Assignments Completed Per Week | 2.63 (2.95) | 3.45 (4.54) | | .026 | .21 |

# Experiment 2: Exploratory analyses on the role of timing

In Experiment 2, students could complete the demographic survey and experimental materials anytime between the end of week 1 and week 5. This flexibility allowed us to reach as many students as possible. It also makes it easier for instructors to implement and integrate the intervention in future courses, enhancing its practical relevance. Further, this variability allowed us to explore whether timing moderated intervention effects.

Students also had flexibility in when to complete the academic confidence survey. This survey was available beginning in the third week, once students had completed the previous material (demographic survey and experimental materials), and remained open until the end of the semester. Thus, we also tested whether the intervention affected when students completed this survey

**Did the timing of intervention completion moderate intervention effects on confidence and/or academic performance?** Examining students’ confidence, we found no evidence for moderation by timing, time (days since course start) × condition, *F*(1,353)=0.46, *p*=.499.

However, analysis of students’ overall grades yielded a significant interaction, *F*(1,458)=9.30, *p*=.002. As seen in Figure S1, the identity-reframing effect on grades was strongest among students who completed the randomized material early in the course. This pattern was driven by a significant interaction for test-performance, *F*(1,457)=14.84, *p*<.001 (Figure S1). No other grade components showed moderation by timing, *p*s>.10. The observation of greater gains among students who completed the randomized materials earlier in the course is consistent with previous research that suggests that psychologically wise interventions in education are often most effective when implemented earlier (e.g., in a course, or in a school transition; Walton & Wilson, 2018; for experimental tests of timing, see Canning et al., 2018; Cook et al., 2012; see also Raudenbusch, 1984). This is when individuals are most liable to be making sense of their experience; early delivery can also help establish adaptive rather than maladaptive recursive cycles that give rise to lasting gains. While correlational, our findings suggest that finding ways to ensure students complete identity-reframing material early in a course could strengthen intervention effects.

**Figure S1.**

*Intervention effects on overall grades (left) and on test performance (right) by time of the completion of experimental materials (Experiment 2).*

**Did the intervention affect when students completed the academic confidence survey?** It did. Low-SES students in the intervention group completed the academic confidence survey roughly three days earlier, around 35 rather than 38 days into the course (intervention: *M*=35.01, *SD*=12,13; control: *M*=37.95; *SD*=13.70), *F*(1, 368)=4-68, *p*=.031. This result is consistent with the theory that the intervention increased low-SES students’ engagement and motivation in the course (e.g., completion of extra credit assignments).

Did this change in timing contribute to effects on students’ reported confidence? It did not. Mediation analysis testing a condition🡪timing🡪 confidence process was not significant, 90%CI=[-.05;.03]. Indeed, there was no correlation between when students completed the academic-confidence survey and their reported confidence, *r*=-.002, *p*=.966.

# Experiment 2: Information on Time Spent on the Learning-Platform

We also obtained data on the time students spent on the learning-platform. Time on the platform did not differ between the intervention (*M*=48.34 hours over the nine course weeks, *SD*=43.07) and control groups (*M*=50.35 hours, *SD*=48.34), *p*=.643. The fact that students in the treatment condition spent no more time on the platform while earning higher grades implies that the identity-reframing exercise helped students study more effectively (see Chen et al., 2017). Using the association between time spent on the platform and overall grades (*b*=.41, *SE*=.01, *β*=.19, *t*=5.27, *p*<.001), we calculated that low-SES students in the control condition would have had to study about five hours longer over the term to achieve the same grades as peers randomized to the 10-minute SES-identity-reframing exercise. If spent on a campus job at the prevailing minimum wage ($12/hour), that five hours would cost students an average of $60 each in earnings.

# References

Canning, E. A., Harackiewicz, J. M., Priniski, S. J., Hecht, C. A., Tibbetts, Y., & Hyde, J. S. (2018). Improving performance and retention in introductory biology with a utility-value intervention. *Journal of Educational Psychology*, *110*(6), 834.

Chen, P., Chavez, O., Ong, D. C., & Gunderson, B. (2017). Strategic Resource Use for Learning: A Self-Administered Intervention That Guides Self-Reflection on Effective Resource Use Enhances Academic Performance. *Psychological Science*, *28*(6), 774–785. https://doi.org/10.1177/0956797617696456

Cook, J. E., Purdie-Vaughns, V., Garcia, J., & Cohen, G. L. (2012). Chronic threat and contingent belonging: Protective benefits of values affirmation on identity development. *Journal of Personality and Social Psychology*, *102*(3), 479.

National Center for Education Statistics. (2023). *Financial Aid—What is the percent of undergraduate students awarded Pell grants?* https://nces.ed.gov/ipeds/trendgenerator/app/answer/8/35
